# Influence of Cryogenic Treatment on the Corrosion Properties of 42CrMo Low Alloy Steel

**DOI:** 10.3390/ma16030899

**Published:** 2023-01-17

**Authors:** Zhi Chen, Chao Li, Hang Su, Yao Huang, Xianguo Yan

**Affiliations:** School of Mechanical Engineering, Taiyuan Science and Technology University, Taiyuan 030024, China

**Keywords:** 42CrMo alloy steel, cryogenic treatment, corrosion resistance, corrosion rate, steel corrosion

## Abstract

In this paper, the effect of deep cryogenic treatment on the corrosion resistance of 42CrMo low alloy steel is investigated and compared with conventional heat-treated counterparts. The low-temperature treatments of the cryogenic process are −120 °C, −160 °C, and −190 °C, respectively. Electrochemical corrosion tests show that the self-corrosion current density of −120 °C, −160 °C and −190 deep-cooled specimens is reduced by 38%, 20% and 30% respectively compared to the usual heat-treated specimens. Scanning electron microscope analysis shows that the precipitation of fine carbides on the surface of the samples treated at −120 °C has improved their corrosion resistance. Electrochemical impedance spectroscopy also shows that the samples treated with −120 °C cryogenic treatment have the smallest corrosion tendency. At a −160 °C deep-cooling process, the precipitated carbide aggregation limits the corrosion resistance of the material. The corrosion resistance of the samples in the −190 °C process group is between the two. The simulation results also express a similar trend to the electrochemical corrosion results.

## 1. Introduction

At present, newly discovered oil and gas fields are mainly high-sulfur oil. As the drilling depth increases, the downhole working conditions are faced with many huge challenges, especially the high-temperature and high-pressure CO_2_, H_2_S, Cl^−^ environment [1,2,3]. As a common material for drill pipe joints in the petroleum industry, the corrosion resistance of 42CrMo alloy steel is particularly important. In recent years, stress corrosion cracking accidents caused by wet H_2_S corrosive media occur frequently [4]. Hydrogen damage will cause metal to crack in deep wells. The essence of its occurrence can be summarized as hydrogen permeation in steel. Hydrogen atoms are accumulated in the gaps between the grainss of the matrix material to form hydrogen, which leads to the formation of bulges inside the material and cracks [5,6]. Accordingly, improved corrosion resistance of the 42CrMo low alloy steel are favorable for improving the lifetime of the drill pipe joints.

At present, low-temperature treatment as a special heat-treatment process is widely used to improve the corrosion resistance of metals and obtain good results [7]. During cryogenic treatment, material is exposed to sub-zero temperatures for a certain period of time in order to improve various material properties, including corrosion resistance [8,9]. Currently there are many studies that show that deep cooling is an effective way to improve the corrosion resistance of materials. Su R.M. found that the cyclic deep cryogenic treatment could improve both the hardness and corrosion resistance of the high-strength 7075 aluminum alloy. After cryogenic treatment, the corrosion resistance is remarkably improved without sacrificing the hardness [10]. Matic has confirmed cryogenic treatment could prolong the lifetime of AISI M35 in corrosive media. The experimental results show that steel’s degradation resistance and functionality can be prolonged by three times when cryogenic treatment is applied [11]. Huang explored a better cryogenic treatment to improve the corrosion resistance of 304LN steel with welds. That paper suggests that it is more appropriate to cool at a rate of 5 °C·min^−1^ and recommends adding a carburizing process before the cryogenic treatment process [12]. Ramesh found through the deep cryogenic treatment process that the steel material enhances the corrosion resistance of structural steel by 95% [13]. Wach has studied the influence of cryogenic treatment on the formation of gas nitride layers on X153CrMoV12 steel. Based on the conventional gas nitriding process, adding cryogenic treatment can increase the hardness, thickness and wear resistance of the nitride layers [14].

The surface of 42CrMo alloy steel will have uniform precipitation of fine carbide after deep cooling treatment [15]. Studies have shown that the precipitation of fine carbides can improve the mechanical properties of materials [16,17]. Currently, most of the literature on research to improve corrosion resistance of 42CrMo alloy steel is based on nitriding and coating to improve corrosion resistance [18,19,20]. Whether these carbides affect the corrosion resistance of 42CrMo alloy steel has not been studied.

Hence, this paper verifies the feasibility of deep cooling treatment to improve the corrosion resistance of 42CrMo and discusses the effect of carbide on corrosion resistance through an electrochemical corrosion test and stress corrosion simulation test. The study provides a reference for improving the drill pipe joint process.

## 2. Materials and Methods

### 2.1. Materials

In this work, the raw 42CrMo steel bar was used in the experiment. Its chemical composition was determined by using an optical emission spectrometer (QSN 750 OBLF, Witten, Germany) according to the specification in the Chinese GB/T 3077 standard and the main components of the material used in Table 1.

**Figure 1:**
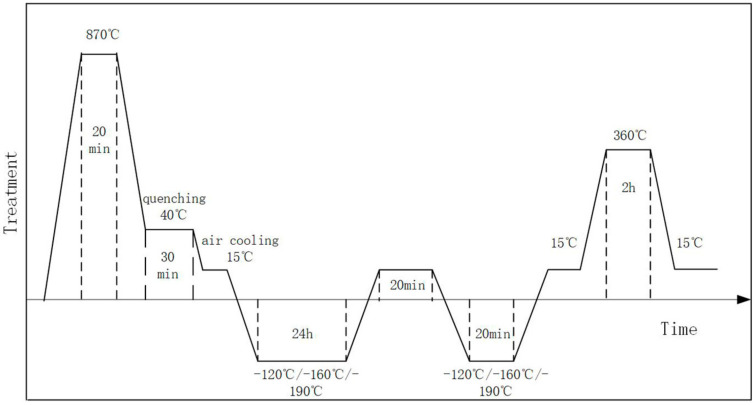
A schematic of process schedules applied for 42CrMo alloy steel.

Workpieces sawed from raw 42CrMo steel bar were first thermos-mechanically treated. The current material heat-treatment manufacturing process is: 870 °C quenching, holding 20 min, 40 °C oil quenching and holding 30 min. Each of the heat-treated workpieces was cut in half, one half for cryogenic treatment and the other half for no cryogenic treatment as a contrast. The whole cryogenic experiment was carried out in a deep-cooling treatment equipment box developed by the research group that could control the cooling, holding and heating process for cryogenic treatment. The parameters of material cryogenic temperature are shown in Table 2. The cryogenically treated workpieces and workpieces without cryogenic treatment were tempered at 360 °C for 2 h. The whole schematic of the process is illustrated in Figure 1.

The 42CrMo alloy steel cryogenic treatment test is a single-factor test. The test variable is the deep-cooling temperature which is set: −120 °C, −160 °C, −190 °C. The material was made into 10 mm × 10 mm × 5 mm specimens by a wire cutting machine (DK7745Z, Wuxi, China) and the treatment process of each specimen is shown in Table 2.

### 2.2. Methods

#### 2.2.1. Electrochemical Corrosion Test

After the cryogenic and tempering processes as mentioned before, the samples were subjected to electrochemical testing to understand the corrosion behavior. The samples were polished with 220, 320, 400, 600, 800, 1000, 1200 grit finish using a rotating wheel mounted with silicon carbide sheet. After that the samples were polished on a linen cloth using 0.5–1.0 μm diamond paste then finished by polishing on velvet cloth. The polished samples were swabbed using deionized water and then with acetone. After that, the samples were dipped in a beaker containing acetone and ultra-sonicated for 3 min. Finally, the samples were swabbed with acetone and deionized water.

The electrochemical corrosion study was conducted in a three-electrode cell assembly using an electrochemical workstation (RST5200F, Zhengzhou, China). The specimen was mounted on a specimen holder with an exposure area of 1 cm^2^. Electrolyte used was 0.1 mol/L of H_2_S solution. The as-polished 42CrMo alloy steel samples were used as working; a saturated calomel electrode and platinum electrode were used as reference and counter electrodes, respectively. A potentiodynamic polarization study was conducted with the applied potential from −0.25~0.5 V with respect to the open circuit potential with a constant scan rate of 0.0005 V/s. Electrochemical impedance spectroscopy is performed at 10^−2^~10^6^ Hz and the amplitude is set to 0.01 V. The mathematical analysis, Tafel fitting Nyquist plots and adjustment of equivalent electrical circuit was performed with ZSimpWin Analysis software (Version 3.21, Ann Arbor, MI, USA).

#### 2.2.2. Microstructure Observation

The sample was polished with sandpaper before observing the microstructure and finally cleaned with ultrasonic waves for 5 min to remove the surface stains. An SEM test was performed with a scanning electron microscope (VEGA3, Shenyang, China) to examine the morphology of specimens.

#### 2.2.3. Hydrogen Sulphide Stress Corrosion Test

A stress corrosion test was performed at room temperature on a slow tensile tester (WDML) made by Xi’an Lichuang company, Xian, China. The experimental medium was hydrogen sulfide solution. The H_2_S solution (0.1 mol/L) was made by passing the H_2_S into distilled water and the pH was controlled to 3.0. The test temperature was 25 °C at room temperature. Before the introduction of H_2_S, high-purity nitrogen was used to remove oxygen for 2 h. After the removal of oxygen, the H_2_S gas was introduced. Stress load was added to the sample at a strain rate of 10^−6^ s^−1^. The size of the specimen is based on Chinese GB/T228-2002: room temperature tensile test method for metallic materials. The exact dimensions of the specimens are given in the subsequent four sections of the simulation.

## 3. Results and Discussion

### 3.1. Effect of Cryogenic Treatment on Microstructure

**Figure 2:**
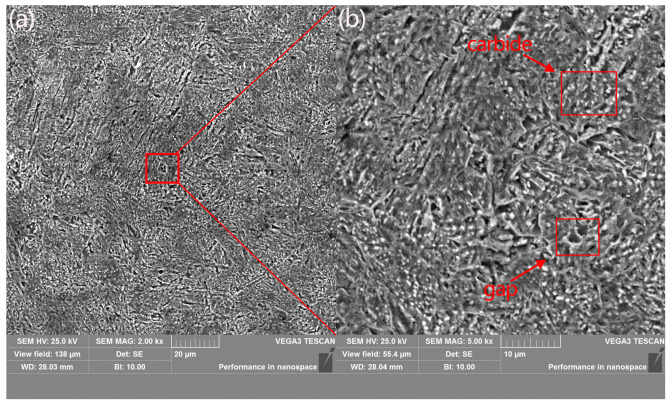
Microstructure of annealed and air-cooled 42CrMo alloy steel (**a**) SEM, magnification ×5000. (**b**) SEM, magnification ×2000.

Figure 2 shows SEM images of the microstructure of the 42CrMo alloy steel through the existing heat treatment. The figure shows the presence of apparent defects and grain gaps on the surface and the presence of white carbide in the microstructure. Observation under a high-magnification SEM reveals that only a small amount of carbide is present on the surface of the material filled with defects. Although the material’s corrosion resistance can be improved to a specific range after this heat treatment process, many defects due to bare leakage can cause rapid damage and corrosion of parts made of this material after a period of use.

**Figure 3:**
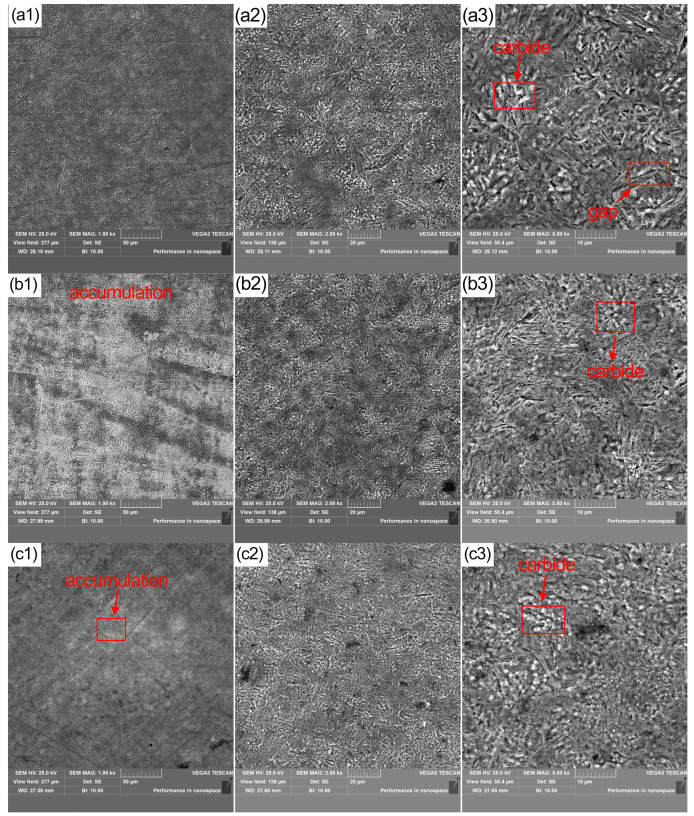
Microstructure of 42MrCo samples after different cryogenic temperature (SEM, magnification (**a1**,**b1**,**c1**) 1000×, (**a2**,**b2**,**c2**) 2000×, (**a3**,**b3**,**c3**) 5000×), (**a**) −120 °C, (**b**) −160 °C, (**c**) −190 °C.

Figure 3 shows the result of SEM test on specimens with cryogenic treatment. In Figure 3a, the specimen had significantly fewer defects and a higher number of diffuse white microscopic carbides on the matrix compared to Figure 2, which was only subjected to conventional heat treatment. The carbide particles were uniformly distributed in the microstructure of the specimens of the process group at −120 °C. Figure 3b,c show the specimens through −160 °C and −190 °C in both process groups, where carbide aggregation was observed. With the increase of deep cooling temperature, the initial precipitation of small carbides continues to aggregate, forming more obvious clusters. Further observation of the microscopic images at higher magnification revealed very small microscopic carbides surrounding the larger white carbide particles in Figure 3c. These clusters form ultra-fine carbides during recovery to room temperature and tempering. These ultra-fine carbides lead to a greater degree of residual stress relief. The precipitation of ultra-fine carbides in grain gaps and defects increases the resistance of the material to micro-cracking and failure deformation, while allowing less hydrogen to enter cracks and thus increasing the corrosion resistance of the material.

It can be seen from Figure 3a that the microstructure of 42CrMo alloy steel has unevenly sized carbides; deep cooling treatment causes the matrix to shrink, resulting in the precipitation and splitting of carbides and appearance of small and uniform carbides on the surface. With increasing deep cooling temperatures, the carbides gradually agglomerate into clusters and become uniform again. The fine carbides fill the material defects, allowing less hydrogen to enter and thus improving corrosion resistance.

### 3.2. Electrochemical Corrosion Test

In Table 3, the shift in corrosion voltage in the positive direction for the specimens after the deep cooling treatment indicates a weakening of the corrosion tendency. The self-corrosion current density decreases by 38 percent at −120 °C, approximately 20 percent at −160 °C, and 30 percent at −190 °C compared to the conventionally annealed air-cooled specimens. Meanwhile, in terms of corrosion depth and corrosion rate, the specimens at −120 °C have the least tendency to corrode. The absolute values of the cathodic Tafel slopes are all greater than the anodic Tafel slopes, indicating that the cathodic reaction of 42CrMo alloy steel in hydrogen sulfide is greater than the anodic reaction. Looking at the overall data in Table 3, the deep cooling treatment process for the material corrosion resistance improves in this order: −120 °C > −190 °C > −160 °C. The corrosion rate of samples from the thermal treatment is much higher than that of samples from other sub-zero temperatures. Within the scope of this study, the material achieved optimal corrosion resistance after cryogenic treatment at −120 °C. The improvement of corrosion resistance of the sample can be attributed to the precipitation of uniform carbide in the sample’s microstructure due to cryogenic treatment.

**Figure 4:**
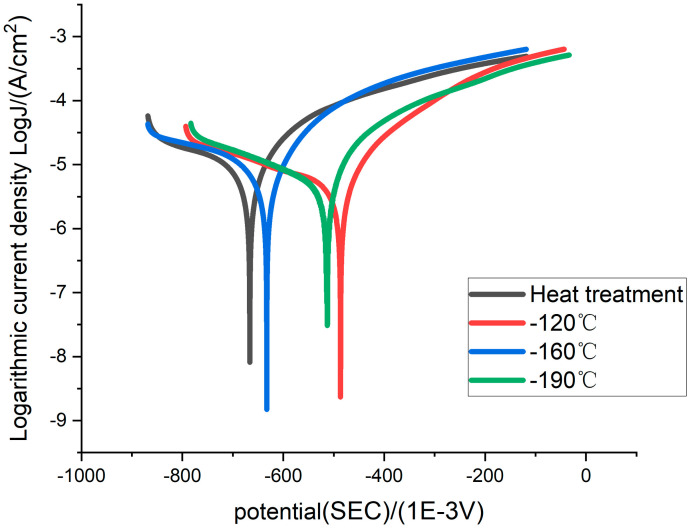
42CrMo alloy steel polarization curve.

As can be seen from Figure 4, the corrosion potential value of samples through cryogenic treatment is shifted to positive values, which indicates an advanced resistance corrosion tendency. After cryogenic treatment, the sample at −120 °C has the lowest corrosion tendency. Combining the corrosion mechanism of steel in wet hydrogen sulfide, we can know the whereabouts of hydrogen produced by hydrogen sulfide corrosion has two parts. One part of the hydrogen ions gain electrons to produce hydrogen gas, and the other part of the hydrogen atoms diffuse into the interior of the 42CrMo alloy steel matrix while gathering at the defects in the 42CrMo alloy steel. Hydrogen atoms entering the matrix will build up at the defect and produce hydrogen gas, eventually causing hydrogen embrittlement and hydrogen damage to the material. The SEM image in Figure 3 shows that the carbide precipitates uniformly on the surface of specimens at −120 °C and −190 °C. Homogeneous carbides adhere to material defects and surfaces within the 42CrMo alloy steel, making it difficult for hydrogen atoms to enter the material. Meanwhile, the increased number of hydrogen atoms in the corrosive medium promotes the cathodic reaction. It accelerates the escape of hydrogen gas, thus allowing the potential to shift positively.

**Figure 5:**
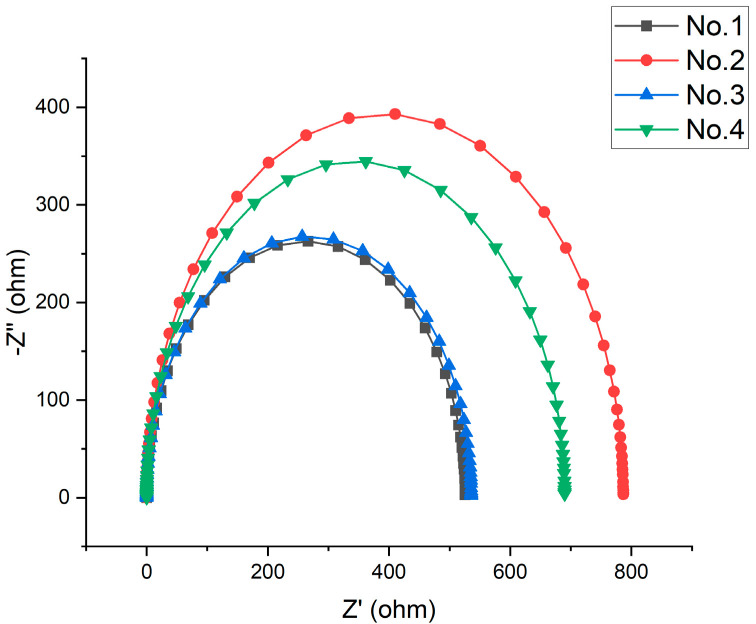
Nyquist diagram of 42CrMo alloy steel electrochemical impedance spectroscopy.

**Figure 6:**
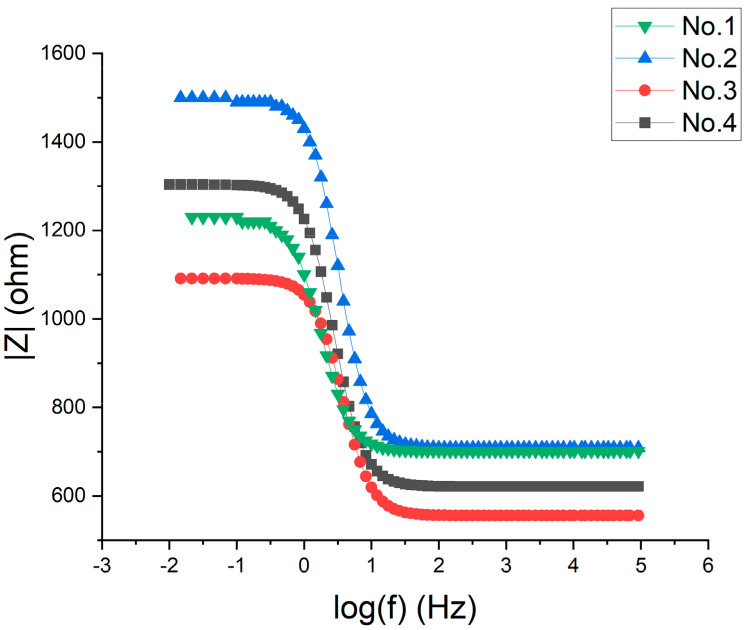
Bode diagram of 42CrMo alloy steel electrochemical impedance spectroscopy.

As can be seen from the Figure 5, the Nyquist diagrams of 42CrMo alloy steel in the hydrogen sulfide liquid after different processes all show a single time constant for the capacitive arc resistance. The radius of capacitive arc resistance increases, then decreases and then increases again with increasing deep cooling temperature. Combined with Figure 6, the increase in the modulus of electrochemical impedance of 42CrMo alloy steel specimens in hydrogen sulfide at −120 °C and −190 °C indicates an increase in corrosion resistance. The improvement in corrosion resistance of deep-cooled −160 °C specimens is very weak.

**Figure 7:**
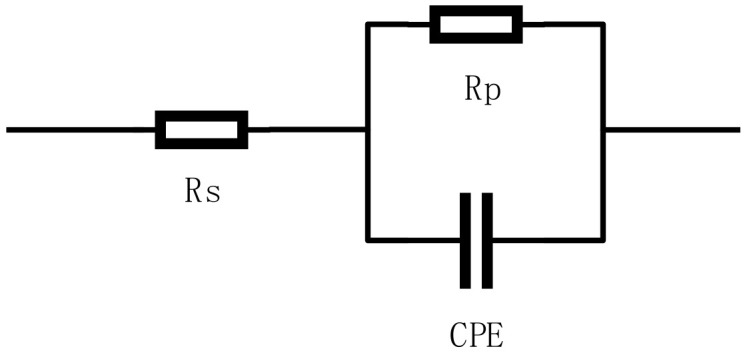
EIS fitting circuit diagram.

The equivalent circuit shown in Figure 7 was used to fit the impedance data obtained in H_2_S solution, including the solution resistance (Rs), the constant phase element representing the double-layer capacitance (CPE), and the charge transfer resistance (Rp). The Rp value represents the impedance of the specimen in solution; the higher the value, the better the corrosion resistance of the material. From the results of the electrochemical impedance spectrum fit in Table 4, it appears that 42CrMo alloy steel has about 1.5 times the resistance after deep cooling at −120 °C compared with after heat treatment. This indicates a minimum tendency to corrode. The corrosion resistance of the material is also improved at 190 °C, and the corrosion trend at −160 °C is almost identical to that of the heat treatment process.

### 3.3. Micro Corrosion Morphology

**Figure 8:**
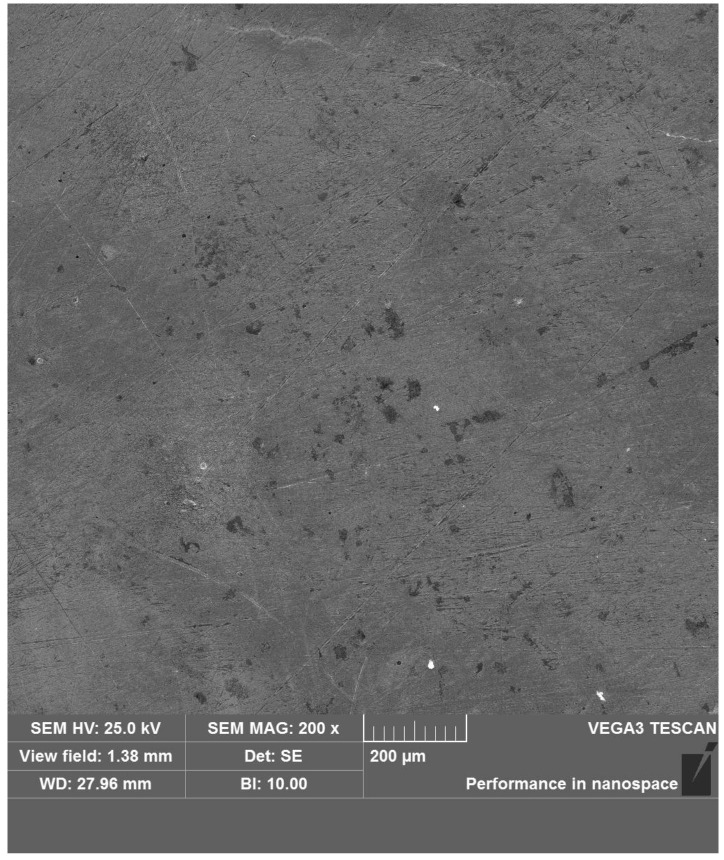
Surface morphology of 42CrMo alloy steel before corrosion (SEM, magnification ×200).

Figure 8 shows the surface of the 42 cm alloy steel without corrosion. It can be seen there are some machining scratches and defect pits on the surface of the material.

**Figure 9:**
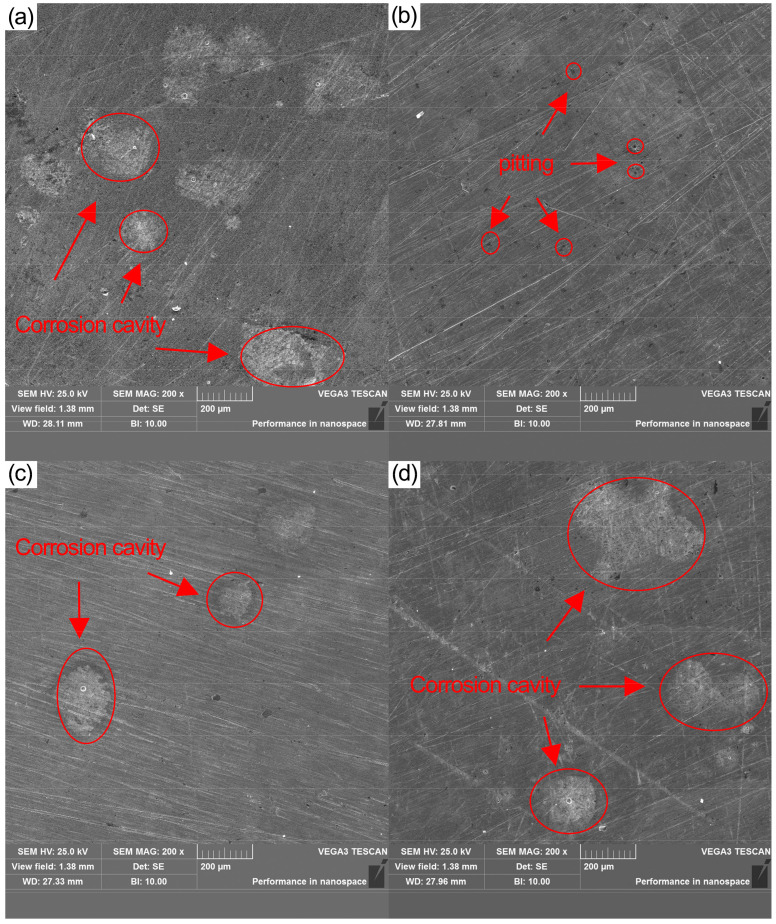
Morphology of different deep cooling temperatures 42CrMo alloy steel after electrochemical corrosion rinsing (SEM, magnification ×200). (**a**) heat treatment (**b**) −120 °C (**c**) −160 °C (**d**) −190 °C.

Figure 9 shows an SEM image of the sample after electrochemical test corrosion and rinsing. Compared with the sample of heat treatment, the surface corrosion morphology of the sample changed significantly after cryogenic treatment. When the temperature is −120 °C, the morphology of the surface can clearly be seen in Figure 9b. In the SEM image, some very minute pits are seen. When the temperature is −160 °C and −190 °C, the no. 3 and no. 4 process group appeared to have more obvious corrosion cavities. Still, the corrosion cavity area, depth, and number are smaller and shallower than the corrosion cavity on the surface of the specimen without deep cooling. In Figure 9c, the corrosion cavity area is small but the overall depth is shallow. In Figure 9d, the corrosion cavity area is large but the overall depth is shallow. This observation indicates that the corrosion resistance of 42CrMo alloy steel is influenced due to the change of microstructure caused by cryogenic treatment. The corrosion tendency is inhibited and the −120 °C process group has the most significant improvement in the corrosion resistance of 42CrMo alloy steel.

In an acidic solution, cementite is the cathode, and ferrite is the anode. Anodic dissolution occurs in the ferrite region, and cathodic reaction occurs in the cementite region. As the ferrite continues to dissolve, the matrix material is destroyed or exfoliated to form cavities on the surface of the 42CrMo alloy steel as shown in Figure 9. In Figure 3b, carbide accumulation is obvious in the −160 °C process group. This case leads to an increase in the electrical potential between cementite and ferrite, making the material corrosion process more severe. At the same time, the dissolution of the ferrite becomes more rapid, resulting in the formation of corrosion cavities as the cementite flakes off the surface of the carbon steel.

## 4. Simulation

**Figure 10:**
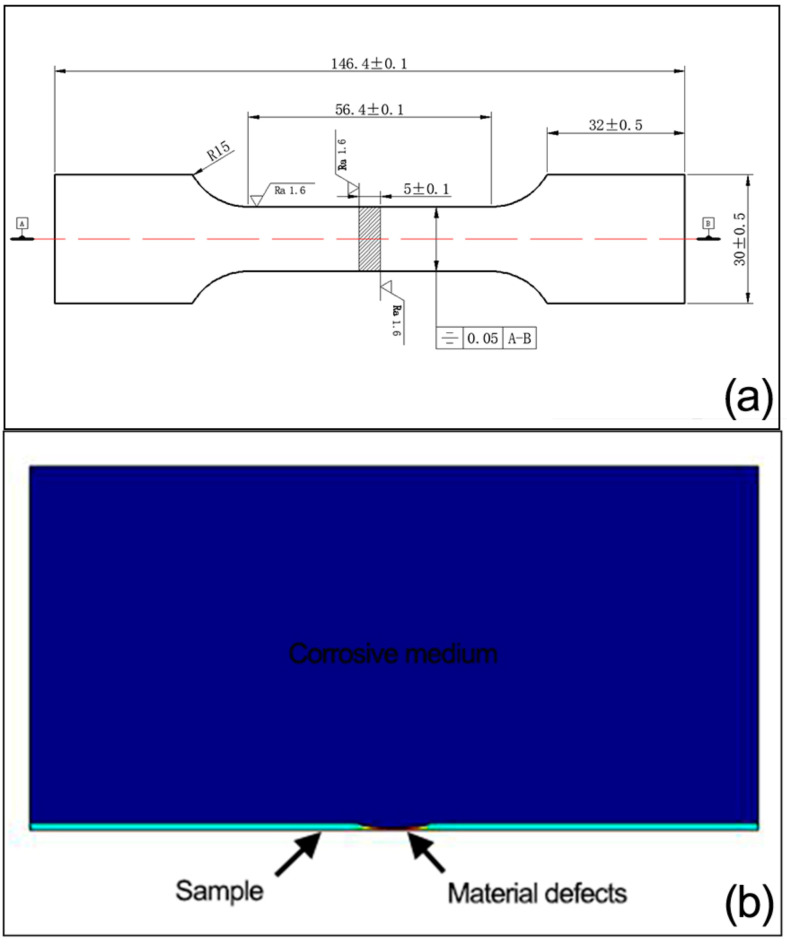
The geometrical model of the steel pipe containing a corrosion defect for simulation (**a**) 3D model (**b**) 2D model.

The simulation of corrosion electrochemical effects on 42CrMo alloy steel was performed using COMSOL Multiphysics 5.4 software. The geometrical model of the steel containing a corrosion defect is shown in Figure 10. The geometry of 42CrMo alloy steel is simplified into a 2D model due to the symmetrical property, as shown in Figure 10b.

The corrosion defect is elliptically shaped, with long semi-axis of 100 mm, and short semi-axis of 11.46 mm.

**Figure 11:**
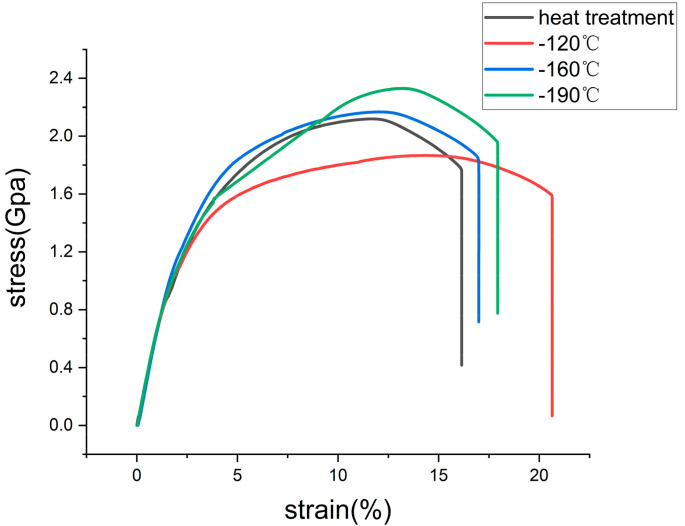
Hydrogen sulfide stress corrosion stress–strain curve of 42CrMo alloy steel at different cryogenic temperatures.

Young’s modulus set 207 × 10^9^ Pa, and the yield strength is taken σ_0.2_ from each curve in Figure 11. The boundary condition of the solution is that the solution boundary is electrically isolated, except the solution/steel interface, which is set as a free boundary. While the left end of the steel model is fixed, the right end is loaded with prescribed displacements of 1.375 mm, 2.75 mm, 3.75 mm, and 4 mm, respectively. The bottom of the model is set as electric grounding. The mesh type used is triangular. A complete mesh consists of 28,542 elements. The maximum and minimum element sizes are 5 mm and 0.1 mm, with a maximum element growth rate of 1.5. A solver of MUMPS (multi-frontal massively parallel sparse) is selected for solution. An elasto-plastic solid stress simulation was performed on model. The isotropic hardening model is selected.

**Figure 12:**
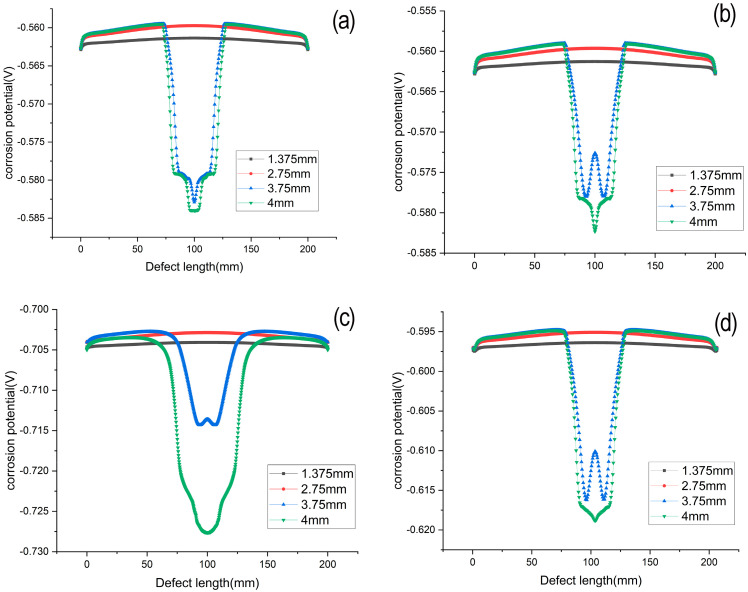
Hydrogen sulfide stress corrosion stress–strain curve of 42CrMo alloy steel at different cryogenic temperatures (**a**) heat temperature (**b**) −120 °C (**c**) −160 °C (**d**) −190 °C.

Figure 12 shows the corrosion potential along the length of the corrosion defect for prescribed displacements of 1.375 mm, 2.75 mm, 3.75 mm, 4 mm, respectively. The corrosion potential has a linear distribution along the bottom of the corrosion defect with 1.375 mm and 2.75 mm. With the applied strain, corrosion potential is shifted negatively. When the prescribed displacement is 3.75 mm, 4 mm, corrosion potential is distributed uniformly, with a more negative potential at the defect center. For the longer tensile strains of 3.75 mm and 4 mm, the entire corrosion defect is observed to be in the plastic deformation.

**Figure 13:**
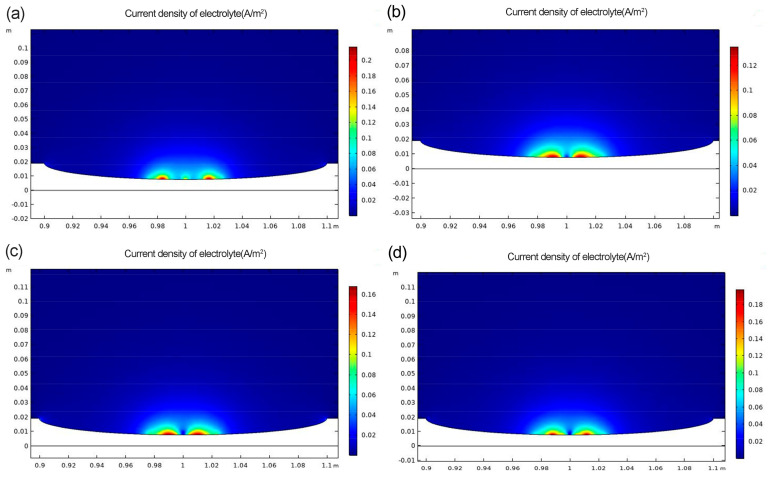
Current density diagram at 3.75 mm tensile displacement (**a**) No. 1 (**b**) No. 2 (**c**) No. 3 (**d**) No. 4.

Figure 13 shows distributions of current density in solution for a corrosion defect at 3.75 mm tensile displacement. The results of the simulation show that the material has the lowest surface current density and the lowest corrosion tendency after a depth treatment of −120 degrees Celsius. The current density on the surface of the material after the deep cooling treatment is lower than the current density of the material after the heat treatment only.

**Figure 14:**
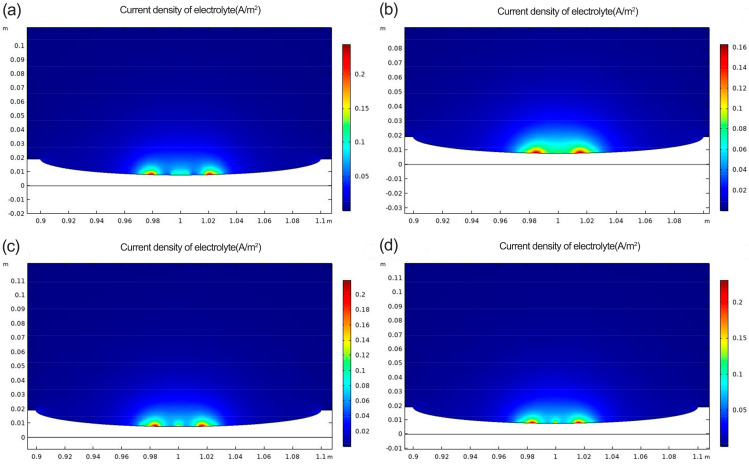
Current density diagram at 4 mm tensile displacement (**a**) No. 1 (**b**) No. 2 (**c**) No. 3 (**d**) No. 4.

Figure 14 shows distributions of current density in solution for a corrosion defect at 4 mm tensile displacement. In Figure 14, the overall current density increases as the tensile displacement increases, resulting in more significant plastic deformation of the material. However, the current density is still minimal in the −120 degrees Celsius process group. Plotting the simulation results in Figure 13 and Figure 14 in Table 5 shows that the specimens in the –120 °C process group have the lowest current density at either 3.75 mm or 4 mm displacement. This indicates a reduction in the amount of corrosive media entering the material and an increase in the corrosion resistance of the material.

## 5. Conclusions

The effects of deep cryogenic treatment on the corrosion resistance of 42CrMo alloy steel were investigated in this work. The main conclusions are shown below.

1. The cryogenic treatment promotes the precipitation and fill of carbides in the 42CrMo alloy steel matrix to grain gaps and defects. With the decrease in cryogenic temperature, surface carbides gradually accumulate and affect the corrosion resistance of materials. After the −120 °C process group, the SEM image displays that the carbide is fine and uniform. The −190 °C process group also improves corrosion resistance, but there is carbide aggregation. After processing at−160 °C, a large number of carbides accumulated, which was not conducive to improving the corrosion resistance of materials.
2. Electrochemical polarization test fitting results show that after the deep cooling process the corrosion current is reduced by 40%, 20% and 30% respectively. The electrochemical impedance spectroscopy results show that 42CrMo alloy steel has the highest impedance value at −120 °C deep cold temperature with the lowest corrosion tendency.
3. In the simulation of stress corrosion with defects, the simulation results also show the most robust corrosion resistance at −120 °C which has a reference value.
4. In this test, the best process group was explored as follows: Quenching at 870 °C for 20 min, oil quenching at 40 °C for 30 min, cryogenic treatment at −120 °C for 24 h, and tempering at 360 °C for 2 h.

## Figures and Tables

**Table 1:** Alloy steel composition of 42CrMo(wt.%).

Element	Mass Percentage	GB/T 3077
C	0.41	0.38–0.45
Cr	0.99	0.90–1.20
Mo	0.18	0.15–0.25
Si	0.21	0.17–0.37
Mn	0.63	0.50–0.80
S	0.0045	<0.0046
P	0.018	<0.035
Fe	balance	-

**Table 2:** Process name and description.

Treatment Process	Cryogenic Treatment (°C)	Cryogenic Time (h)	Sample Number
Annealed-Air cooled	-	-	No. 1
Annealed-Air cooled +cryogenic	−120	24	No. 2
Annealed-Air cooled +cryogenic	−160	24	No. 3
Annealed-Air cooled +cryogenic	−190	24	No. 4

**Table 3:** Corrosion parameter values of polarization curve.

Specimen	Corrosive Potential (SEC) (mV)	Self-Corrosive Current Density (A/cm^2^)	Cathode Tafel Slope (V/dec)	Anode Tafel Slope (V/dec)	Corrosion Rate (g/m^2^h)	Corrosion Depth (mm/year)
No. 1	−666.4	3.16 × 10^−6^	−0.08	0.06	3.22 × 10^−2^	3.61 × 10^−2^
No. 2	−486.5	1.99 × 10^−6^	−0.12	0.06	2.73 × 10^−2^	3.06 × 10^−2^
No. 3	−632.8	2.51 × 10^−6^	−0.08	0.06	4.09 × 10^−2^	4.50 × 10^−2^
No. 4	−513.7	2.24 × 10^−6^	−0.09	0.06	2.87 × 10^−2^	3.21 × 10^−2^

**Table 4:** EIS test fitting data.

Treatment Process	Rs (Ω·cm^2^)	Rp (Ω·cm^2^)
Annealed-Air cooled	2.7	525.4
Annealed-Air cooled +cryogenic treatment (−120 °C)	3	815.6
Annealed-Air cooled +cryogenic treatment (−160 °C)	2.8	525.7
Annealed-Air cooled +cryogenic treatment (−190 °C)	2.9	625.6

**Table 5:** Current density of each process sample under strain 3.75 mm and 4 mm.

Displacements	3.75 mm				4 mm			
Cryogenic treatment	-	−120 °C	−160 °C	−190 °C	-	−120 °C	−160 °C	−190 °C
Current density (A/m^2^)	0.20	0.11	0.20	0.21	0.21	0.13	0.20	0.15

## Data Availability

Not applicable.
